# Perceived stigma of COVID-19 patients in Shanghai, China, in the third year of the pandemic: a cross-sectional social impact survey

**DOI:** 10.1186/s12889-023-16604-9

**Published:** 2023-09-04

**Authors:** Ziru Deng, Ausma Bernot, Sara E. Davies

**Affiliations:** 1 School of Public Health, Li Ka Shing Faculty of Medicine, The University of Hong Kong, Hong Kong, China; 2 School of Criminology and Criminal Justice, Griffith University, 176 Messines Ridge Road, Mt Gravatt, QLD 4122 Australia; 3 School of Government and International Relations, Griffith University, Nathan, QLD 4111 Australia

**Keywords:** COVID-19, Stigma, Social Impact Scale, China

## Abstract

**Introduction:**

Social stigma associated with Covid-19 infection has been reported around the world. This paper investigates the level of self-reported perceived stigma among people infected with COVID-19 in Shanghai, China, in the third year of the pandemic to determine changes in perceived stigma and individual level variables associated with perceived stigma.

**Methods:**

We conducted a self-reported two-part online survey (*n* = 144 responses) by employing a convenience sampling method of COVID-19 patients in Shanghai. The first part of the survey collects sociodemographic information of the respondents and the second part outlines 24 items of the Social Impact Scale (SIS), which measures individual level factors associated with stigma, namely social rejection, financial insecurity, internalized shame, and social isolation. We ran Wilcoxon signed-ranks test, Kruskal–Wallis test, and linear regression analysis to assess the levels of perceived stigma differences.

**Results:**

The study finds that the overall level of self-reported stigma during the COVID-19 lockdowns in Shanghai in 2022 was at a lower level than that compared to the self-reported perceived stigma study in Wuhan in 2020. In Shanghai, the severity of the disease and hospitalization length had most impact on financial insecurity and feelings of social isolation. These experiences were not gendered. Recovery measures, including economic considerations, need to pay particular attention to those who experienced severe disease.

**Supplementary Information:**

The online version contains supplementary material available at 10.1186/s12889-023-16604-9.

## Introduction

On 28 February 2022, an outbreak of COVID-19 began in Shanghai, China. 5 April 2022 Shanghai reported 268 new cases (totaling 13,086 active cases across the Shanghai municipality) [1] and entered a whole-city Covid lockdown after a few weeks of local area lockdowns that failed to contain the spread of the virus [2]. The full lockdown was lifted on 1 June 2022, nearly two months after hard city-wide lockdowns. Following the removal of the lockdown, the infection case numbers continued climbing. Partial lockdowns were again instilled in the city just a few days after.

Lockdowns and social isolation immensely disrupt people’s normal lives [3]. COVID-19 spread not inflicts negative health outcomes directly related to COVID-19, but also has negative social impacts. For example, Gan et al. found that province-wide lockdowns had long-term psychological impacts on the 59 million individuals who remained in Hubei, the epidemic epicenter during the pandemic [4]. Although Wuhan’s 72-day lockdown remains the longest one in the country to date, Shanghai lockdowns were found to have had a high stress impact among residents in Shanghai [5]. Through the currently public research, Shanghai lockdowns were shown to have caused healthcare interruptions in vulnerable populations [6], emotional overload as well as family and community conflicts, and psychiatric medicine shortage, among known impacts [7]. By reviewing gender disaggregated data, studies have found that women experienced higher levels of anxiety [8] and posttraumatic stress symptoms [9] across China. Stigma and discrimination have also become the challenges that would increase the suffering of patients both physically and mentally during and after the lockdowns. The classic definition of stigma by Goffman is an individual’s possession of some attribute that causes the individual to be classified within a “discredited” social category [10]. In social science research, Link and Phelan conceptualize stigma as the co-occurrence of its components—labeling, stereotyping, separation, status loss, and discrimination [11]. Stigma has always been a concern in the context of epidemics, but the way it unfolds may vary due to different social contexts [12]. Perceived stigma is about individual’s awareness of actual or potential social disqualification due to certain characteristics. The perceived stigma from infectious diseases could lead to increased risks of depression, anxiety, and posttraumatic stress disorder among survivors [13]. Previous studies have shown that individuals from Hubei have experienced strong perceived stigma, which is associated with greater psychological distress and the additional challenge of stigmatization and discrimination [14]. However, to our best knowledge, the perceived stigma among the COVID-19 survivors in Shanghai is understudied.

In the early stages of the epidemic between February and March 2020, Lin and associates [15] found that among residents of Wuhan who contracted COVID-19 perceived social stigma was at a moderate to severe level. Age, marital status, occupation, and disease severity had impacts of the levels of perceived stigma. Wuhan residents suffered stigma at both the individual and community levels [16]. Other regions in China also found higher levels of self-reported stigma among people who had contracted COVID-19 against healthy populations [17]. Stigma is a serious barrier to health seeking behavior. Tracking stigma experiences is important to ensure effective multi-level interventions [18]. Although the new prevention and control measures announced on 7 December 2022 do not include lockdowns [19], investigating the perceived stigma of COVID-19 patients can provide lessons for public health crises and insights into mental health services in the future. In this research, we employ an online survey method to test perceived stigma amid people who contracted COVID -19 in Shanghai between February and June 2022. In doing so, we analyze the self-reported perceived stigma in Shanghai and compare it with the previous study among the COVID-19 patients in Wuhan. Additional insight in this study is gender-disaggregation of survey data.

## Methods

To measure the stigma perceived from COVID-19, the study employs a 24-item Social Impact Scale (SIS), which measures social rejection, financial insecurity, internalized shame, and social isolation in four subscales [20]. The SIS is widely used among patients with medical conditions or infectious diseases. The Chinese version of the SIS has been validated with good psychometric properties and used for multiple populations [21]. Additionally, a previous study has used the Chinese version of the SIS to evaluate the social stigma levels of asymptomatic COVID-19 carriers in China [22]. The self-reported survey in this study proceeds in two parts: the first part collects sociodemographic information of the respondents and the second part outlines 24 items of the SIS (see Additional file 1 for details). The item is measured with a 4-point Likert scale. The higher SIS score means a lower perceived stigma. In this study, the SIS demonstrated strong reliability and construct validity, with a Cronbach﻿’s α coefficient of 0.962 and a Kaiser–Meyer–Olkin coefficient of 0.950 (*p* < 0.005).

### Patient and public involvement

The participants recruited in this study consented to participate in the survey voluntarily and anonymously. The survey information sheet and participant consent form included contact information of the research team, which participants could use to request information about the study or its publications. To maintain the anonymity of the participants, we did not include participant’s contact information as part of the study design.

### Data collection

The survey inclusion criteria were the date of COVID-19 contraction (between February 28 and June 30, 2022), residence in Shanghai during the time of COVID-19 contraction, and age (over 18 years old).

The data collection for the survey was facilitated through a two-pronged strategy of collecting responses through Qualtrics survey company (*n* = 107) and the researchers’ own networks (*n* = 57). Qualtrics managed the survey administration by utilizing market research panels to gather respondents for the study. These respondents were invited through various methods, including email invitations, in-app notifications, and SMS notifications, ensuring a diverse pool of participants. To maintain data quality, Qualtrics employed ID verification and their data quality management (e.g., ISO-certified panels, in-house Research Services quality program). The second response collection strategy utilized researchers’ networks. We posted the open questionnaire invitation on their own WeChat accounts and under the hashtags “Shanghai”, “COVID-19”, and “quarantine” on Weibo and Little Red Book social media platforms. Additionally, one researcher was invited to a WeChat group of patients who had been quarantined together in a Fangcang shelter hospital in Shanghai by a known COVID-19 patient. The inclusion criteria of the survey (i.e., time of contraction, Shanghai-based residence location, and minimum respondent age of 18) were stated both in the participant information sheet and consent form that were displayed on the first page of the survey. Participants were prompted to review and confirm the key inclusion details by clicking an “I agree” button in the survey.

The two-pronged data collection yielded 211 responses. After the removal of incomplete responses, we included 164 complete responses, out of which 20 were deleted due to incorrectly entered age and COVID-19 contraction date. For data validity, we controlled the speed of survey completion and straight-lining. Any responses that took less than 96 seconds (a minimum time achieved in the testing of the survey stage of 10 people) to complete or had identical answers for all 24 items in the SIS scale were excluded. We were also able to verify the geographical latitude and longitude of the responses included, all of which were in the Shanghai area. This study relies on self-reported survey data, which has advantages in terms of quickly gathering a large number of responses but also has limits, the most pertinent of which to this survey are honesty and introspective ability and recall bias.

A total of 144 responses were analyzed following data cleaning (the Additional file 2 “SIS study raw data” shares the data used in this research study).

### Statistical analysis

We used the Statistical Package for Social Sciences (SPSS 28.0) software for data analysis. Frequency and median were used to describe the general information of the survey sample and the perceived stigma level of COVID-19 patients. A series of Wilcoxon signed-ranks test, Kruskal–Wallis H test, and linear regression were applied to assess the differences in the SIS total and four category scores related to the sociodemographic variables. To ensure comparability with the 2020 Lin and associate’s study from COVID-19 patients in Wuhan, we tested all statistics with a two-sided test and *p*-value with the confidence interval of 95%.

## Results

### Sociodemographic characteristics

Table 1 shows that the majority of the 144 respondents were residents of Shanghai (97.2%) and were not living alone (84.0%). Among all the respondents, 47.2% were male and 52.8% were female; 37.5% were younger than 30 years old, 47.9% were aged between 30 and 50, and 14.6% were aged over 50. The education level of the respondents was generally high: 63.2% of the respondents had a bachelor’s degree and 16.7% had postgraduate education. Patients with asymptomatic and mild symptoms accounted for 35.4% respectively, and over half of the respondents experienced hospitalization (including medical quarantine facilities) of over 2 weeks (56.3%). Due to the grueling quarantine policies in place during most of the lockdown period, only 11 out of 144 respondents (7.6%) were able to quarantine at home. Due to a requirement to test negative prior to release from the hospital, some patients experienced exceedingly long hospitalization times: 7 respondents reported spending 30 days and 1 spent 36 days in the hospital.

**Table 1:** Survey results of stigma with different socio-demographic characteristics

Sociodemographic characteristic	Number	Ratio	SIS score Median (Q1, Q3)	*χ* ^*2*^*/Z*	*P* value
**Gender**					
Male	68	47.2%	66 (53.25,81.75)	-0.576^a^	0.564
Female	76	52.8%	65 (53.5,77)		
**Age**					
≤ 29	54	37.5%	68.5(56.5,80.25)	1.926	0.382
30 ~ 50	69	47.9%	63(50.5,81)		
≥ 50	21	14.6%	66(55,75.5)		
**Education**					
High school or below	9	6.2%	57 (50,64.5)	4.033	0.258
Junior college	28	13.9%	65 (45,77.75)		
Bachelor’s degree	91	63.2%	66 (56,81)		
Master’s degree or above	24	16.7%	70.5 (53.75,81.75)		
**Occupation**					
Migrant worker/farmer	4	2.8%	60 (54,70.5)	0.928	0.921
Civil servant/public institution	17	11.8%	65 (53.5,80.5)		
Enterprise or freelancer	98	68.1%	66 (53,81)		
Student	22	15.3%	71 (52,79.5)		
Retired	3	2.1%	64 (63,-)		
**Single residence**					
Yes	23	16.0%	65 (51,75)	-0.445^a^	0.657
No	121	84.0%	66 (53.5,81)		
**Marital status**					
Single or widowed or divorced or separated	50	34.7%	68.5 (54.25,79.5)	-0.886^a^	0.376
Married or in domestic partnership	94	65.3%	64.5 (53,80.25)		
**Dependents under 18**					
Yes	78	54.2%	63.5 (52.5,78.25)	-1.861^a^	0.063
No	66	45.8%	69.5 (56.75,82)		
**Monthly household income (CNY)**					
≤ 10,000	29	20.1%	63(54,72.5)	4.609	0.100
10,000–20,000	55	38.2%	64(51,77)		
≥ 20,000	60	41.7%	70(58,82.75)		
**Resident of Shanghai**					
Yes	140	97.2%	66(54.25,80.75)	-1.246^a^	0.213
No	4	2.8%	56.5(37.25,69)		
**Severity of COVID-19**					
Asymptomatic	51	35.4%	68(55,81)	14.595	**0.002**
Mild	51	35.4%	68(63,82)		
Moderate	40	27.8%	58(45,72.75)		
Severe	2	1.4%	38.5(37,-)		
**Days of hospitalization**					
< 14 (2 weeks)	63	43.8%	71(59,82)	-2.554^a^	**0.011**
≥ 14 (2 weeks)	81	56.3%	62(51,78.5)		

^a^Z value

### Overall perceived stigma on COVID-19

The average SIS score of the 144 participants was 65.72 (63.00, 68.44). The SIS score was higher compared to the 2020 findings from Wuhan, which averaged at 57.37 (*n* = 122). The higher score from the Shanghai data suggests that residents self-reported less perceived stigma as compared to Wuhan residents.

The post-hoc power analysis implies that the study’s power is at its maximum, indicating high sensitivity to detect effects. A standard alpha value of 5% was used. The mean difference was found to be 8.35, determined through average population values from Wuhan (57.37, *N* = 122) compared with the average value of our study results (65.72). The standard deviation for the sample is 16.5, resulting in a Cohen’s effect size (d) of 0.85. Considering our sample size is 144, the null hypothesis presupposes a T-distribution with 143 degrees of freedom. In contrast, the alternative hypothesis presumes a Noncentral-T distribution with 143 degrees of freedom and a non-centrality parameter of 6.073. According to a two-sided test, we infer that the power of this test is 1. This power analysis provides meaningful input regarding the statistical robustness and precision of our study.

### Single-factor analysis

The key significant factor in self-reported overall SIS scores (including all four SIS categories of *social rejection*, *financial insecurity*, *internalized shame*, *social isolation*) was the severity of COVID-19 infection (*p* = 0.002) and days in hospitalization (*p* = 0.011), with other variables and disaggregated gender data not confirming statistical significance (see Table 1).

The aggregate SIS score for people who spent 14 days or more in the hospital following their diagnosis was significantly lower as compared to those who spent fewer days hospitalized. A further analysis of significance against the disaggregated SIS scores, as shown in Table 2, shows that financial insecurity stigma scores were most impacted by days of hospitalization (*p* = 0.003), dependents under 18 (*p* = 0.008), and monthly household income (*p* = 0.014). Social isolation stigma scores were most impacted by dependents under 18 (*p* = 0.049), severity of COVID-19 (*p* = 0.009) and days of hospitalization (*p* = 0.012).

**Table 2:** Disaggregated SIS scores across variable categories of significance

Sociodemographic characteristic	Social Rejection			Financial Insecurity			Internalized Shame			Social Isolation		
	**Median**	***χ*** ^**2**^**/Z**	**P**	**Median**	***χ*** ^**2**^**/Z**	**P**	**Median**	***χ*** ^**2**^**/Z**	**P**	**Median**	***χ*** ^**2**^**/Z**	**P**
Dependents under 18												
Yes	24(18.75,30)	-1.632^a^	0.103	7(5,10)	-2.670^a^	**0.008**	13(11,16)	-0.801^a^	0.423	20(16,23)	-1.971^a^	**0.049**
No	26(22,32)			9(6,10.25)			14(11,17)			21(17.75,25)		
Monthly household income (CNY)												
≤ 10,000	25(19,28)	3.644	0.162	8(5,9)	8.480	**0.014**	13(11,15)	2.888	0.236	18(16.5,21)	3.382	0.184
10,000–20,000	23(19,28)			7(5,9)			13(10,17)			20(17,23)		
≥ 20,000	27.5(22,31.75)			9(6,10.75)			14.5(11,17)			21(16,25)		
Severity of COVID-19												
Asymptomatic	27(20,32)	14.388	**0.002**	8(6,10)	7.763	0.051	14(11,17)	12.853	**0.005**	21(16,25)	11.615	**0.009**
Mild	26(23,31)			8(6,10)			14(12,17)			21(18,24)		
Moderate	22(16,27)			6.5(5,9)			11.5(10,14.75)			18(15.25,21)		
Severe	15(14,-)			5(5,5)			7(7,7)			11.5(11,-)		
Days of hospitalization												
< 14 (2 weeks)	26(22,32)	-1.887^a^	0.059	9(6,10)	-2.933^a^	**0.003**	14(12,17)	-2.383^a^	0.017	21(18,25)	-2.521^a^	**0.012**
≥ 14 (2 weeks)	24(18,29)			7(5,9)			12(10,16)			19(16,23)		

^a^Z value

Furthermore, we used the Bonferroni method to perform the pairwise multiple comparison analysis for different levels of the severity of COVID-19 symptoms and monthly household income against the total and disaggregated SIS scores, as shown in Table 3. The results indicate that patients with mild COVID-19 symptoms perceived less overall stigma and experienced less internalized shame stigma compared with those with moderate and severe symptoms (*p* = 0.018 and 0.043 for total SIS scores, *p* = 0.042 and 0.033 for internalized shame scores, respectively). In terms of social rejection, patients with mild symptoms perceived less stigma than those with moderate symptoms (*p* = 0.013). Additionally, patients earning more than 20,000 CNY per month perceived less financial insecurity than those with a monthly household income of 10,000 to 20,000 CNY (*p* = 0.011).

**Table 3:** Multiple comparisons of COVID-19 severity and income on the perceived stigma of COVID-19 patients

Category SIS	Pairwise comparisons	Mean Difference	95% CI	P (Bonferroni corrected)
**Total SIS**	**Severity of COVID-19**			
	Mild vs. Moderate	10.072	(1.13,19.02)	0.018
	Mild vs. Severe	31.147	(0.62,61.67)	0.043
**Social rejection**	**Severity of COVID-19**			
	Mild vs. Moderate	4.258	(0.61,7.91)	0.013
**Financial insecurity**	**Monthly household income (CNY)**			
	≥ 20,000 vs. 10,000–20,000	1.379	(0.24,2.51)	0.011
**Internalized shame**	**Severity of COVID-19**			
	Mild vs. Moderate	2.075	(0.05,4.1)	0.042
	Mild vs. Severe	7.275	(0.36,14.19)	0.033

### Gender disaggregated analysis

Studies from other countries that measured self-reported stigma via SIS scores found that gender disaggregated data revealed self-reported SIS differences between men and women [23]. Gender disaggregated data from our study, although reporting slightly lower self-reported values for women (see Table 4 for details), showed no significant difference in the SIS score observed between men (mean = 66.54) and women (mean = 64.99). We had included an option for respondents to choose a gender option other than “male” or “female” but received no responses. Further significance tests of gender disaggregated data across variables (education, occupation, employment, residence and marital status, dependents, income, disease severity) show no significance.

**Table 4:** Four categories of SIS scores disaggregated by gender

SIS Scores	**Male (*n* = 68)**	**Female (*n* = 76)**	**Statistics**	
	**Median (Q1, Q3)**	**Median (Q1,Q3)**	**Z**	**P**
Overall stigma	66 (53.25,81.75)	65 (53.5,77)	-0.576	0.564
Social rejection	25 (19,32)	25 (20.25,29)	-0.587	0.557
Financial insecurity	7.5 (5.25,10)	7.5 (6,9)	-0.711	0.477
Internalized shame	13.5 (11,17)	13 (10,16.75)	-0.026	0.979
Social isolation	21 (16.25,23.75)	20 (16.25,23)	-0.848	0.396

### Linear regression analysis

Following the identification of significant independent variables and their impact on the aggregated and disaggregated SIS scores, we conducted linear regression analyses to detail the explanatory variables. The increased severity of COVID-19 symptoms and length of hospitalization negatively impacted the overall SIS score: moderate symptoms *p* = 0.032; severe symptoms *p* = 0.022, compared to the asymptomatic patient group; hospitalization of over 14 days *p* = 0.039 compared to the patients who were hospitalized for less than 14 days (see Table 5 for details).

**Table 5:** Linear regression analysis of the total SIS scores

***Independent variables***	b^a^	β^b^	***t***	***P***	**Adjusted R**^**2**^	**F**	***p***
(constant)	70.405		27.618	**< 0.001**	0.104	5.150	**< 0.001**
Severity of COVID-19 (ref. asymptomatic)							
Mild	2.287	0.066	0.738	0.462			
Moderate	-7.225	-0.197	-2.170	**0.032**			
Severe	-26.358	-0.187	-2.322	**0.022**			
Hospitalization length (ref. less than 14 days)							
Over 14 days	-5.547	-0.167	-2.083	**0.039**			

^a^b: unstandardized regression coefficients;

^b^β: standardized regression coefficients

We divided the four categories of SIS scores into dependent variables. Independent variables, which had significant impact on the perceived stigma in the previous single-factor analysis, were included as independent variables to conduct linear regression analyses. Namely, for social rejection and internalized shame scores, we included the severity of COVID-19; for financial insecurity scores, we included dependents under 18, monthly household income, and hospitalization length; for social isolation scores, we included dependents under 18, severity of COVID-19 and hospitalization length (see Table 6 for details).

**Table 6:** Linear regression analysis of category SIS scores

***Category SIS***	***Independent variables***	b^a^	β^b^	***t***	***P***	**Adjusted R**^**2**^	**F**	***p***
**Social rejection**	(constant)	25.706		28.451	< 0.001	0.081	5.210	**0.002**
	Severity of COVID-19 (ref. asymptomatic)							
	Mild	0.627	0.045	0.491	0.624			
	Moderate	-3.631	-0.242	-2.664	**0.009**			
	Severe	-10.706	-0.187	-2.302	**0.023**			
**Financial insecurity**	(constant)	8.287		16.484	< 0.001	0.031	2.512	0.061
	Dependents under 18 (ref. no dependent)							
	Have dependents under 18	-1.081	-0.210	-2.633	**0.009**			
	Monthly household income (ref. ≤ 10,000 CNY)							
	10,000 ~ 20,000	0.131	0.025	0.231	0.818			
	≥ 20,000	1.275	0.245	2.322	**0.022**			
	Hospitalization length (ref. less than 14 days)							
	Over 14 days	-1.034	-0.200	-2.506	**0.013**			
**Internalized shame**	(constant)	13.745		27.389	< 0.001	0.074	4.790	**0.003**
	Severity of COVID-19 (ref. asymptomatic)							
	Mild	0.529	0.068	0.746	0.457			
	Moderate	-1.545	-0.187	-2.041	**0.043**			
	Severe	-6.745	-0.213	-2.611	**0.010**			
**Social isolation**	(constant)	22.001		26.041	< 0.001			
	Dependents under 18 (ref. no dependent)							
	Have dependents under 18	-2.002	-0.199	-2.469	**0.015**	0.063	4.192	**0.007**
	Severity of COVID-19 (ref. asymptomatic)							
	Mild	0.970	0.092	1.027	0.306			
	Moderate	-1.663	-0.148	-1.651	0.101			
	Severe	-9.021	-0.210	-2.604	**0.010**			
	Hospitalization length (ref. less than 14 days)							
	Over 14 days	-1.479	-0.146	-1.821	0.071			

^a^b: unstandardized regression coefficients

^b^β: standardized regression coefficient

For the social rejection and internalized shame scores, COVID-19 severity was found to have statistically significant impacts. Compared to asymptomatic patients, severe and moderate patients were more likely to report social rejection and internalized shame: social rejection scores were statistically significant for both moderate patients (*p* = 0.009) and severe patients (*p* = 0.023); internalized shame scores were also statistically significant for moderate patients (*p* = 0.043) and severe patients (*p* = 0.010). Patients with mild COVID-19 symptoms reported no significant difference from asymptomatic patients in the social rejection and internalized shame categories.

Patients who have dependents under 18 and were hospitalized over 14 days experienced significantly more financial insecurity compared to those without dependents under 18 (*p* = 0.009) and shorter hospitalization length (*p* = 0.009). Additionally, monthly household income was found to be a significant predictor of financial insecurity, with patients whose monthly household income was more than 20,000 CNY reporting less financial insecurity compared to those whose monthly household income less than 10,000 CNY (*p* = 0.022). In terms of social isolation scores, severe patients (*p* = 0.010) and patients with dependents under 18 (*p* = 0.015) also reported more social isolation, as compared to asymptomatic patients and patients without dependents under 18.

## Discussion

This research study finds that the overall level of self-reported stigma during the COVID-19 lockdowns in Shanghai in 2022 (average at 65.72 out of 96) was lower than that in Wuhan in 2020 (57.37) [15]. Our study also indicates a lower level of overall perceived stigma compared to COVID-19 patients in Chongqing in 2020 [17], who reported an average score at 70.2 out of 96 (given the reverse Likert scale setting in their study, with higher scores indicating a higher level of perceived stigma). Similarly, a study in Germany in 2020 reported a lower level of perceived stigma of COVID-19 survivors in 2020, with an aggregate SIS mean score was 36.29 (with lower scores indicating a lower level of perceived stigma) [24]. Considering the disease context in the third year of the pandemic, the lower level of perceived stigma in Shanghai may correlate to the milder and shorter Omicron variant infection and the surge of asymptomatic patients. Additionally, Shanghai also reported a better result in a previous nationwide survey of psychological distress levels during the COVID-19 pandemic [25], which may be related to the city’s public health system, considered to be one of most efficient in China and having implemented effective public health strategies during the early COVID-19 outbreak [26].

Lin and associates [15] had reported impacts across occupations, age, marital status, and severity of disease in the Wuhan study. In Shanghai, the severity of the disease and the length of hospitalization had the most impact on the total SIS scale. This study did not show any significant difference in stigma based on gender, level of education, or residence status, which is consistent with the 2021 Jiang and associates’ nationwide study [16].

Quarantine or hospitalization may interrupt individuals’ work or professional activities with no advanced planning [27], resulting in anticipated earnings wilted. During previous epidemics, financial loss was found to be one of the stressors of negative psychological effects [28], which is confirmed by the reported financial insecurity results in this study. People with higher income are likely to feel less financial insecurity during the pandemic. However, the financial impact of COVID-19 significantly negatively affected people who were hospitalized for 14 days or longer and have dependents under 18. In June 2022, the Shanghai municipal authorities announced subsidies for employers under the policy called “Shanghai Action Plan for Accelerating Economic Recovery and Revitalization” [29], which supported companies in providing employment for such recent university graduates and people who had been unemployed for three months or more. Our study indicates that municipal authorities should also consider providing direct financial subsidies for people who are hospitalized for 14 days or longer and people who have underage dependents. In situations where quarantine is deemed necessary, authorities should quarantine residents for no longer than required, ensure sufficient food and medical supplies, and provide financial support for loss of income throughout the quarantine period [30].

Feelings of social rejection and internalized shame were strongest for people who had more severe symptoms of COVID-19. During the lockdowns, people who tested positive for COVID-19 were taken to centralized medical quarantine facilities, colloquially known as “Fangcang hospitals”. According to the March 2022 guidelines from the National Health Commission, patients were not discharged until consecutively testing negative twice (at an interval of 24 h) [30]. As such, long-lasting quarantines and the uncertainties caused by these kinds of isolation were likely to affect feelings of being stigmatized, as was similarly identified in qualitative studies in Shandong province [31] and Finland [32].

As early as 2020, China’s National Health Commission recognized that a mental health crisis is likely to accompany COVID-19 crisis and encouraged localities across China to run free-of-charge mental health hotlines [7]. Shanghai municipality’s hotline logged a 79% increase of incoming calls in March at the beginning of the citywide lockdown, as compared to the previous month [7]. Despite the availability of this counselling hotline at the time of survey, reported social isolation scores were high. Chen and associates found that during the pandemic in Shanghai, even among the asymptomatic COVID-19 carriers, people who suffered from stigma were more likely to feel trapped and decadent, thus affecting depression [22]. This points to the need for additional mental health support measures (such as support groups and psychological interventions), particularly for people who are impacted by long hospitalization times and/or experienced severe COVID-19 symptoms. Perceived social support and government support, such as providing adequate resources and clearly communicated public health messages, would in turn protect people’s mental health as well during the COVID-19 quarantine and lockdowns [4, 33]. Moreover, special attention should also be paid to patients who were caring for underage dependents while hospitalized or in quarantine as they reported high social isolation scores.

Amongst the social demographic groups surveyed, gender disaggregated data did not reveal significant differences in self-reported SIS results, suggesting that the Shanghai COVID-19 lockdown impacts were not significantly gendered. Gender did not appear to determine feelings of social isolation, separation from children, or financial impact.

This study is a rapid investigation to describe the level of perceived stigma among the COVID-19 patients during the Shanghai lockdown in 2022. The study reports some limitations and future direction. Due to the convenience sampling method, the sample size of farmers and migrant workers, which was a key group reporting disparities in the Wuhan study, is small (*n* = 4) and not representative in our study. The online survey method may not capture a diverse and broader socio-demographic population, particularly individuals with low income and low education levels, resulting in lower representativeness of this study. Also, since stigma often continues for some time after quarantine, or even after the containment of the outbreak [18], further qualitative inquiries into the post-COVID era and the disparities behind the lighter effects of self-reported stigma could explore what factors reduce self-reported stigma over time. Self-reported survey data also may contain recall bias and carries the risk of respondents not answering the questions honestly.

## Conclusion

Our survey reveals that individual self-perceived stigma associated with COVID-19 infection might have reduced over time due to a greater understanding of the infection, strain variation, and medical interventions available to reduce severity of infection. The survey also reveals a relationship between individual perception of stigma and severity of disease experience. Individualized perception of stigma and severity of disease may be an important consideration when devising long-term care interventions for individuals recovering from COVID-19 infection, and those diagnosed with long COVID [34]. These experiences do not appear to be gendered and therefore there is a need to ensure that all populations have opportunity to resolve their perception and experience of stigma.

Importantly, this survey found that health related stigma crosscuts with economic and social vulnerability. There will be a need for health and social welfare systems to consider socio-economic harms from COVID-19 infection, especially the experience of hospitalization, quarantine, and severity of disease. These experiences may increase individual risk of financial insecurity and mental trauma. This means that governments need to prepare for populations who, depending on the size of population who experienced severe COVID-19 infection and prolonged hospitalization, may continue to experience financial, social, and emotional stress.

### Supplementary Information

**Additional file 1.** COVID-19 social stigma survey.

**Additional file 2.** SIS study raw data.

## Data Availability

The survey questionnaire used in this study is included as the Additional supplementary file 1. All data analysed in this study is de-identified and included in the Additional supplementary file 2.
