# Supplementary material for: Perceived stigma of COVID-19 patients in Shanghai, China, in the third year of the pandemic: a cross-sectional social impact survey
Source: BMC Public Health. 2023 Sep 4;23:1709. doi: 10.1186/s12889-023-16604-9 (PMC10476326; doi:10.1186/s12889-023-16604-9)
Supplement: Supplementary file 1 — Additional file 1. COVID-19 social stigma survey. [file 12889_2023_16604_MOESM1_ESM.pdf]

COVID-19 Social Stigma Survey

新型冠状病毒肺炎患者污名化感知调查表

Dear Respondent,

This is an online Social Impact Survey for Shanghai residents who had contracted COVID-19 between Feb 28 and June 30, 2022. The purpose of this survey is to understand the effects of COVID-19 on social exclusion, economic discrimination, intrinsic shame, and social isolation. Your participation is voluntary, and you are free to withdraw from the survey at any time. Your decision to participate or withdraw from this research will in no way impact you. Your name and other personal information that could identify you will be removed or de-identified in publications or presentations resulting from this research.

This project 2020/229 has been approved by the Human Research Ethics Committees of Simon Fraser University and Griffith University in compliance with the requirements of the Australian National Statement of Ethical Conduct in Human Research, [updated 07/12/2021].

Thank you for your cooperation!

尊敬的新型冠状病毒肺炎患者朋友：

您好！

本调查是针对 2022 年 2 月 28 日至 6 月 30 日期间感染 COVID-19 新型冠状病毒肺炎的上海居民的社会影响网络调查。本次调查的目的是为了解 COVID-19 患者面临的社会排斥、经济不安全感、内在羞耻感和社会隔离的影响。您参与本次调查是自愿的，并且您可以在任何时候无条件地自由退出本调查。您的退出不会有任何后果。您的个人信息（包括名字以及其他能进行身份识别的信息）会被严格保密，并且不会出现在本调查产生的各种类型的研究成果中（包括出版物及学术会议）。本项目 2020/229 已获得西蒙弗雷泽大学和格里菲斯大学人类研究伦理委员会的批准，符合澳大利亚国家人类研究伦理行为声明[2021 年 12 月 7 日更新]的要求。

感谢您的合作!

Before you begin the survey, please confirm the following survey requirements:

√ I have had Covid-19 between Feb 28 and June 30, 2022 and was residing in Shanghai at that time

√ I am 18 years old or older

√ I have read the Participant Information Sheet and Consent Form, and I agree to take part in this survey

在开始调查之前，请您确认以下信息：

√ 我在 2022 年 2 月 28 日至 6 月 30 日期间曾感染过新型冠状病毒肺炎，且当时居住在上海

√ 我已年满 18 岁

√ 我已阅读调查背景信息和知情同意书，并同意参与本次调查

Sociodemographic Characteristics

一、社会人口学特征

1. Gender: ① Male ② Female ③ Other, please 您的性别：①男 ②女 ③ 其它，请注明 _____
2. Age:____您的年龄：____岁
3. Education: ① primary school or below ② middle school ③ high school ④ junior college ⑤ bachelor’s degree ⑥ master’s degree or above 您的文化程度：①小学或未接受正式教育 ② 初中 ③高中 ④专科学校 ⑤ 本科 ⑥ 硕士及以上
4. Occupation: ① migrant worker or farmer ② civil servant or public institution ③ enterprise or freelancer ④ student ⑤ retired 您的职业：①民工或务农 ②公务员或事业单位 ③企业或自由职业者 ④学生 ⑤退休人员
5. Are you a resident of Shanghai? ① Yes ② No
   您是否长期居住在上海？①是 ②否
   5.1. If you do not live in Shanghai for a long time, you visit Shanghai for the following purposes: ① travel ② visit relatives ③ work ④ medical services ⑤other purposes, please specify 如果您并非长期居住在上海，您此次来上海目的是：
   ①旅游 ② 探亲 ③ 工作 ④ 就医 ⑤ 其它，请注明_________
6. Are you living alone? ①Yes ② No 您是否自己独居？①是 ②否
7. Monthly household income: ① <3,000 yuan ② 3,000-5,000 yuan ③ 5,001-10,000 ④10,001-20,000 yuan ⑤>20,000 yuan 您的家庭月收入：① ① 3000 元以下② 3000-5000 元③ 5001-10000 元④10001 元-20000 元⑤ 20000 元以上
8. Marital status: ①single ② married or in domestic partnership ③ widowed ④ divorced or separated 您的婚姻状况：①单身②已婚或同居③丧偶④离婚或分居
9. Do you have any dependents who are 18 years of age or younger? ① Yes ② No
   您是否有 18 岁或者 18 岁以下的孩子？①是 ②否
   9.1 If yes, how many? 如是，您有几个？

Covid-19 Investigation

二、新型冠状病毒肺炎病情调查

10. The Date of last Covid-19 diagnosis (day, month): _____
    您上一次确诊阳性的日期：____月____日
11. Have you ever admitted to a hospital or mobile cabin hospital? ①Yes ② No
    在此次患病期间，您是否曾在医院或方舱医院进行医治或隔离？①是 ② 否
    11.1 If yes, Duration of last Covid-19 hospitalization (days): ____
    若是，您的住院时长为____（天）
12. What Covid-19 symptoms did you experience?* ① asymptomatic ②mild ③ moderate ④severe ⑤critical
    您新型冠状病毒肺炎的感染程度*
    ①无症状感染 ②轻型症状 ③普通型症状 ④重型症状 ⑤危重型症状

*Asymptomatic: Positive SARS-CoV-2 test; no symptoms.

Mild: Mild symptoms (e.g. fever, cough, or change in taste or smell); no dyspnea; no radiographic evidence.

Moderate: Clinical or radiographic evidence of lower respiratory tract disease; oxygen saturation≥93%

Severe: Oxygen saturation＜93%; respiratory rate≥30breaths/min; lung infiltrates＞50%.

Critical: Respiratory failure, shock, and multiorgan dysfunction or failure.

*无症状感染：无临床症状，呼吸道等标本新型冠状病毒病原学检测阳性者。

轻型症状：轻型患者可表现为低热、轻微乏力、嗅觉及味觉障碍等, 影像学未见肺炎表现。

普通型症状：具有发热、呼吸道感染等临床表现，影像学可见肺炎表现。

重型症状：符合氧饱和度≤93%；气促，RR≥30 次/分；肺部影像学显示病灶明显进展＞50%之一。

危重型症状：出现呼吸衰竭；休克；合并其他器官功能衰竭情况之一。

Social Impact Survey

三、疾病社会影响调查

Please tick "√" according to your true feelings since your diagnosis.

① Strongly agree ② Agree ③ Disagree ④ Strongly disagree

请根据您确诊新型冠状病毒肺炎以来的真实感受，选择最符合您的答案打"√"

① 非常同意②同意③不同意④非常不同意

**Social Impact Scale items 疾病社会影响量表条目**	**①Strongly agree 非常同意**	**②Agree 同意**	**③ Disagree 不同意**	**④ Strongly disagree 非常不同意**
**Social Rejection 社会排斥**				
1. My employer/co-workers have discriminated against me. 我的老板或同事曾因我的感染而歧视我。				
2. Some people act as though I am less competent than usual. 有些人认为我的能力不如感染前了。				
3. I feel I have been treated with less respect than usual by others. 与感染前相比，我觉得我不如从前那样受人尊重了。				
4. I feel others are concerned they could "catch" my illness through contact like a handshake or eating food I prepare. 我感觉别人会担心因为和我的接触，而感染了我的病，如与我握手或吃我准备的食物。				
5. I feel others avoid me because of my illness. 我感觉别人因为我的感染而回避我。				
6. Some family members have rejected me because of my illness. 一些家人因为我的感染而排斥我。				
7. I feel some friends have rejected me because of my illness. 我感觉有些朋友因为我的感染而排斥我。				

8. I encounter embarrassing situations as a result of my illness.<br>由于我的感染，我曾遇到一些令我难堪的状况。				
9. Due to my illness others seem to feel awkward and tense when they are around me.<br>由于我的感染，别人在我周围时他们似乎感到尴尬和紧张。				
**Financial Insecurity 经济不安全感**				
10. I have experienced financial hardship that has affected how I feel about myself.<br>疫情期间，我经历过经济困难，这影响了我的自我感受。				
11. My job security has been affected by my illness.<br>我的感染影响了我的工作保障。				
12. I have experienced financial hardship that has affected my relationships with others.<br>疫情期间，我经历过经济困难，这影响了我的人际交往。				
**Internalized Shame 内在羞耻感**				
13. I feel others think I am to blame for my illness.<br>我觉得别人认为我感染这个病应该怪我自己。				
14. I do not feel I can be open with others about my illness.<br>我觉得我无法对别人公开我感染过这个病。				
15. I fear someone telling others about my illness without my permission.<br>我害怕有人未经我的允许告知别人我感染过这个病。				
16. I feel I need to keep my illness a secret.<br>我觉得我需要为我的感染保密。				
17. I feel I need to keep my illness a secret.<br>我觉得我感染这个病至少有部分该怪我自己。				
**Social Isolation 社会隔离**				
18. I feel set apart from others who are well. 我觉得我与其他健康的人不同。				
19. I have a greater need than usual for reassurance that others care about me. 我比感染前更需要确保别人关心我。				
20. I feel lonely more often than usual.<br>我比感染前更常感到孤单。				

21. Due to my illness, I have a sense of being unequal in my relationships with others.<br>由于我的感染，我在与他人的人际交往中感到不平等。				
22. I feel less competent than I did before my illness.<br>我感觉我的能力不如感染前。				
23. Due to my illness, I sometimes feel useless.<br>由于我的感染，有时候我觉得自己没有用。				
24. Due to my illness, I sometimes feel useless.<br>我外表上的变化已经影响了我的人际交往。				
